# Critically evaluated key points on hereditary medullary thyroid carcinoma

**DOI:** 10.3389/fendo.2024.1412942

**Published:** 2024-06-11

**Authors:** Daqi Zhang, Nan Liang, Hui Sun, Francesco Frattini, Chengqiu Sui, Mingyu Yang, Hongbo Wang, Gianlorenzo Dionigi

**Affiliations:** ^1^ Division of Thyroid Surgery, The China-Japan Union Hospital of Jilin University, Jilin Provincial Key Laboratory of Surgical Translational Medicine, Jilin Provincial Precision Medicine Laboratory of Molecular Biology and Translational Medicine on Differentiated Thyroid Carcinoma, Changchun, China; ^2^ Division of Surgery, Istituto Auxologico Italiano IRCCS (Istituto di Ricovero e Cura a Carattere Scientifco), Milan, Italy; ^3^ Department of Pathophysiology and Transplantation, University of Milan, Milan, Italy

**Keywords:** thyroid, medullary thyroid carcinoma, hereditary, calcitonin, CEA, men, diagnosis, pathology

## Abstract

Medullary thyroid carcinoma (MTC) accounts for only 3% of all thyroid carcinomas: 75% as sporadic MTC (sMTC) and 25% as hereditary MTC (hMTC) in the context of multiple endocrine neoplasia type 2 (MEN2). Early diagnosis is possible by determining the tumour marker calcitonin (Ctn) when clarifying nodular goitre and by detecting the mutation in the proto-oncogene RET in the MEN2 families. If the Ctn level is only slightly elevated, up to 30 pg/ml in women and up to 60 pg/ml in men, follow-up checks are advisable. At higher levels, surgery should be considered; at a level of > 100 pg/ml, surgery is always advisable. The treatment of choice is total thyroidectomy, possibly with central lymphadenectomy. In the early stage, cure is possible with adequate surgery; in the late stage, treatment with tyrosine kinase inhibitors is an option. RET A mutation analysis should be performed on all patients with MTC. During follow-up, a biochemical distinction is made between: healed (Ctn not measurably low), biochemically incomplete (Ctn increased without tumour detection) and structural tumour detection (metastases on imaging). After MTC surgery, the following results should be available for classification in follow-up care: (i) histology, Ctn immunohistology if necessary, (ii) classification according to the pTNM scheme, (iii) the result of the RET analysis for categorisation into the hereditary or sporadic variant and (iiii) the postoperative Ctn value. Tumour progression is determined by assessing the Ctn doubling time and the RECIST criteria on imaging. In most cases, “active surveillance” is possible. In the case of progression and symptoms, the following applies: local (palliative surgery, radiotherapy) before systemic (tyrosine kinase inhibitors).

## Introduction

**Figure 1:**
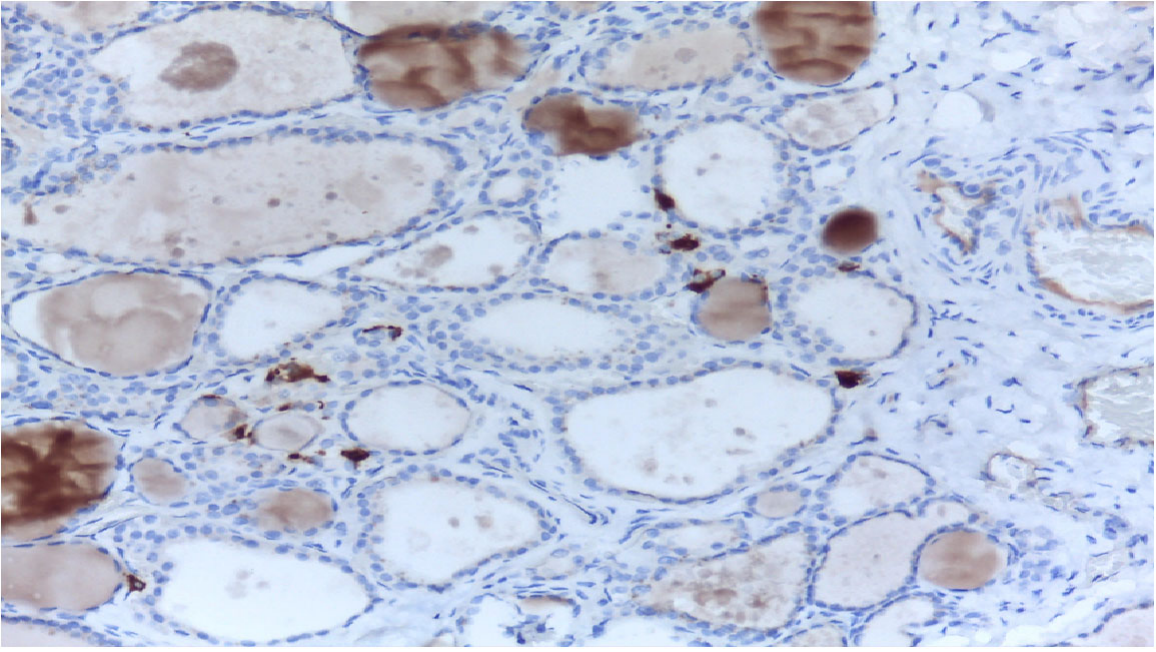
MTC in histology. C-cells and MTC are usually found in the posterior upper third of the lateral lobes.

Medullary thyroid carcinoma (MTC) is a neuroendocrine tumour and represents a malignant transformation of the C-cells of the thyroid gland (1) (**Figure 1**). It secretes calcitonin (Ctn) and carcinoembryonic antigen (CEA) and occurs more frequently in families (multiple endocrine neoplasia type 2, MEN2). Early diagnosis through consistent Ctn determination in the clarification of a multinodular goitre and adequate primary surgery is the only possibility for a possible cure of MTC (2). MTC is not involved in iodine metabolism and does not store iodine, so radioiodine therapy is not possible (3). MEN2 is caused by a germline mutation of the protooncogene RET (1–3). In MEN2, early diagnosis of gene carriers before the occurrence of carcinoma can be achieved by molecular genetic testing of the RET protooncogene as part of family screening (1–3). Overall, MTC has a relatively favourable prognosis with a relatively good quality of life. In many cases, a risk-adapted strategy of active surveillance is possible for patients who have not recovered. Patients in the stage of symptomatic progressive distant metastasis can be treated with tyrosine kinase inhibitors if palliative local measures are not sufficient (1–3). The 5- and 10-year survival rates are 80% and 60% respectively (4).

## MTC detection

**Figure 2:**
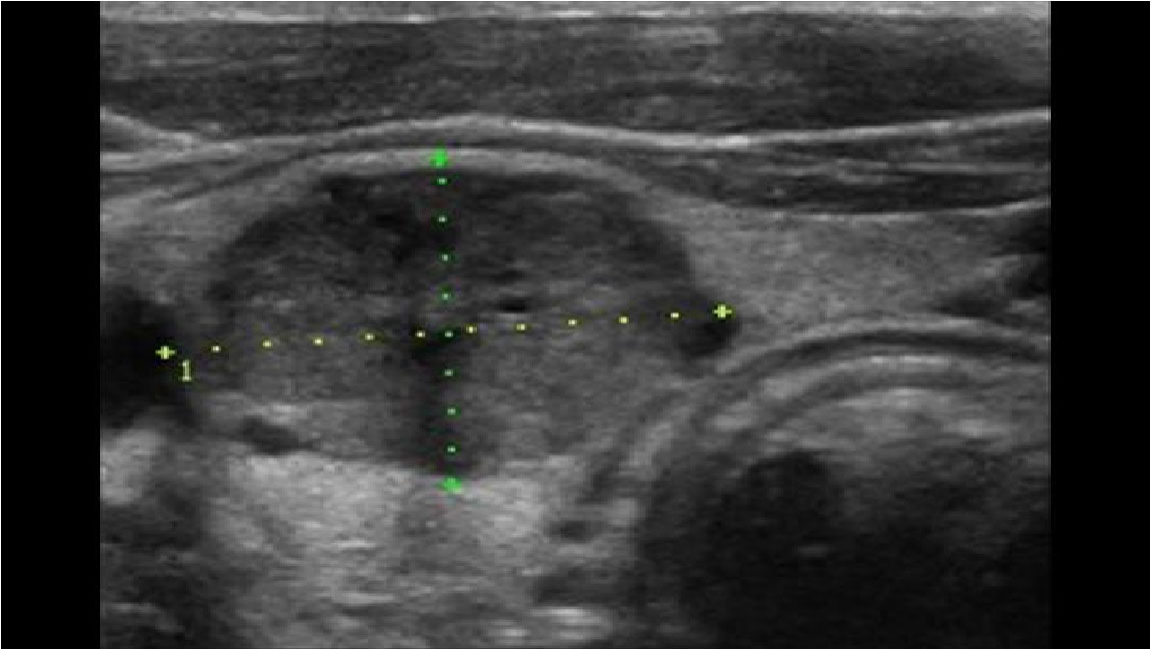
MTC in ultrasound: solid inner content, ovoid to round, hypoechoic (50%), calcifications (50–80%).

MTC is often diagnosed as an incidental finding on histological examination of a specimen after thyroid surgery or on examination of a thyroid nodule (5) (**Figure 1**). A suspicious nodule on thyroid ultrasound (hypoechoic, microcalcifications, blurred margins, deeper than wide), suspicious cervical lymph nodes, a suspicious fine needle aspiration and an elevated Ctn value point in the right direction (**Figure 2**). Patients in the 4th and 5th decade of life are affected. In the advanced metastatic stage, therapy-resistant diarrhoea may occur. The rare MTC should be taken into consideration when investigating an increase in CEA (1, 3, 6).

## Ctn - the tumour marker

Ctn, a polypeptide hormone secreted by thyroid C-cells correlates with the tumour mass and is a sensitive and specific tumour marker for the early detection and follow-up of MTC. Slightly elevated Ctn values are found in C-cell hyperplasia, which can be a precursor to hereditary MTC, but is also observed as a “benign” concomitant symptom in other thyroid diseases (4–7). Ctn levels greater than 50 -100 ng/L are strictly related to the diagnosis of MTC and MTC without Ctn expression and secretion is rare (5, 6).

On the other hand the interpretation of moderately high Ctn levels is more difficult as it can be influenced by many conditions as sex, age, drugs, short half-life of Ctn.

## Ctn screening in multinodular goitre

The routine determination of calcitonin in the investigation of multinodular goitre enables early diagnosis and thus improves the chances of cure and therefore the prognosis. Ctn screening in nodular goitre is advocated by the European Thyroid Society (6, 7). In the past, the following limitations were reasons for the low acceptance of screening:

- The diagnostic uncertainty of only slightly elevated Ctn values due to a lack of specificity
- The non-comparability of old Ctn tests
- The increased effort involved in stimulating the Ctn with pentagastrin or calcium,
- The inconsistently defined threshold values for a pathological stimulation test.

However, with the development of newer sensitive and more specific fully automated Ctn tests [chemiluminescence immunoassays with a sensitivity of 0.5 pg/ml and gender-specific upper reference values (8, 9)], basal Ctn values have become more important for screening for nodular goitre. With preoperative Ctn values of > 100 pg/ml, MTC is present in almost 100% of cases, whereas with Ctn values of 10–20 pg/ml, the detection rate for MTC is only 5% (10). Taking sensitivity and specificity into account, current studies show gender-specific cut-off values for basal calcitonin to recommend surgery for suspected MTC of around 30 pg/ml for women and around 60 pg/ml for men.

The grey zone for Ctn is 20–30 pg/ml for women and 30–60 pg/ml for men; here 6–13% of small MTCs were missed (11). Elevated Ctn levels are more indicative of MTC and surgery should be recommended. Surgical measures can cure almost 100 of MTCs with a Ctn value of up to 100 pg/ml (12).

Carcinoembryonic antigen (CEA)

Carcinoembryonic antigen is a marker expressed by the neuroendocrine cells of the gastrointestinal tract and is elevated in 60–70% of MTC. CEA is not a specific marker for diagnosis of MTC. A raise of CEA may have some prognostic utility as its levels may be related to the progression and the extent of the disease (13).

## RET proto-oncogene - germline mutations in MEN2

A germline mutation in the RET proto-oncogene can be detected in around 25–30% of patients with MTC as part of a hereditary variant (MEN2). In the hereditary form of MTC, the causative mutations have been characterised in almost all families, mostly point mutations in 8 different exons of the proto-oncogene RET. The molecular genetic detection of these mutations makes it possible to cure MTC in families by planning an early thyroidectomy. The optimal timing for thyroidectomy in gene carriers of an RET mutation is now recommended based on the risk classification of the specific RET mutation into moderate, high and highest risk for early development of MTC (early or late penetrance) (**Table 1**) (1). The Ctn value supplements and defines the individually planned prophylactic thyroidectomy at an early stage, so that in the best case an additional lymphadenectomy is not necessary. Depending on the genotype, up to 50% of MEN2 patients develop pheochromocytomas in the course of their lives, usually after the occurrence of an MTC. The pheochromocytomas can be multifocal and bilateral (14). Patients with RET 634 and 918 mutations are particularly affected, less frequently with mutations in exons 13–15. Up to 10% of MEN2 patients can develop genotype-dependent primary hyperparathyroidism; patients with an RET -918 mutation are not affected. Other extrathyroidal manifestations, e.g. ganglioneuromatosis in MEN2B or lichen amyloidosis interscapularis, are also genotype-dependent. Not all families can be recognised on the basis of family history: *De novo* mutations occur in a low percentage, the patient does not know any other family members, there are only small families or the penetrance of late-manifesting hMTC is low. A germline mutation RET was found in 12% of apparently sporadic MTC.

**Table 1:** Risk stratification and treatment of patients with MEN2 depending on the RET mutation [from: Wells et al. 2015 (1)].

Codone	533, 609, 611, 618, 620, 630, 631, 768, 790, 804, 891,	634	918
*ATA (2015)* (2)	Moderate	High	Highest
*MEN2 subtype*	FMTC/MEN2	MEN2A	MEN2B
*Occurrence of MTC*	Usually 5 years and older	Often before the age of 5	Often in the first year of life
*Time of prophylactic* *thyroidectomy*	as soon as Ctn > 10 pg/ml rises/ mostly in adolescence	before the age of 5	as soon as possible, in the 1st year of life
*Screening pheochromocytoma*	from the age of 16, annually	from the age of 11, annually	from the age of 11, annually
*Screening primary hyperparathyroidism*	from the age of 16, annually	from the age of 11, annually	–

MTC, medullary thyroid carcinoma; FMTC, familial MTC; ATA, American Thyroid Association; Ctn, calcitonin.

## RET proto-oncogene - somatic mutations

A mutation in the RET gene, usually M918 T, is often detectable in the tumour tissue of sporadic MTC. Interestingly, this mutation is less frequent in smaller tumours, more frequent in larger tumours and most frequent in lymph nodes and distant metastases, suggesting that this mutation is not the primary event in tumour development. In tumour tissue in which no RET mutation is found, a RAS mutation can often be detected. Detection of the somatic RET mutation has prognostic significance and is a prerequisite for treatment with selective RET tyrosine kinase inhibitors (LOXO-292, BLU-667) (15).

## Surgery

**Figure 3:**
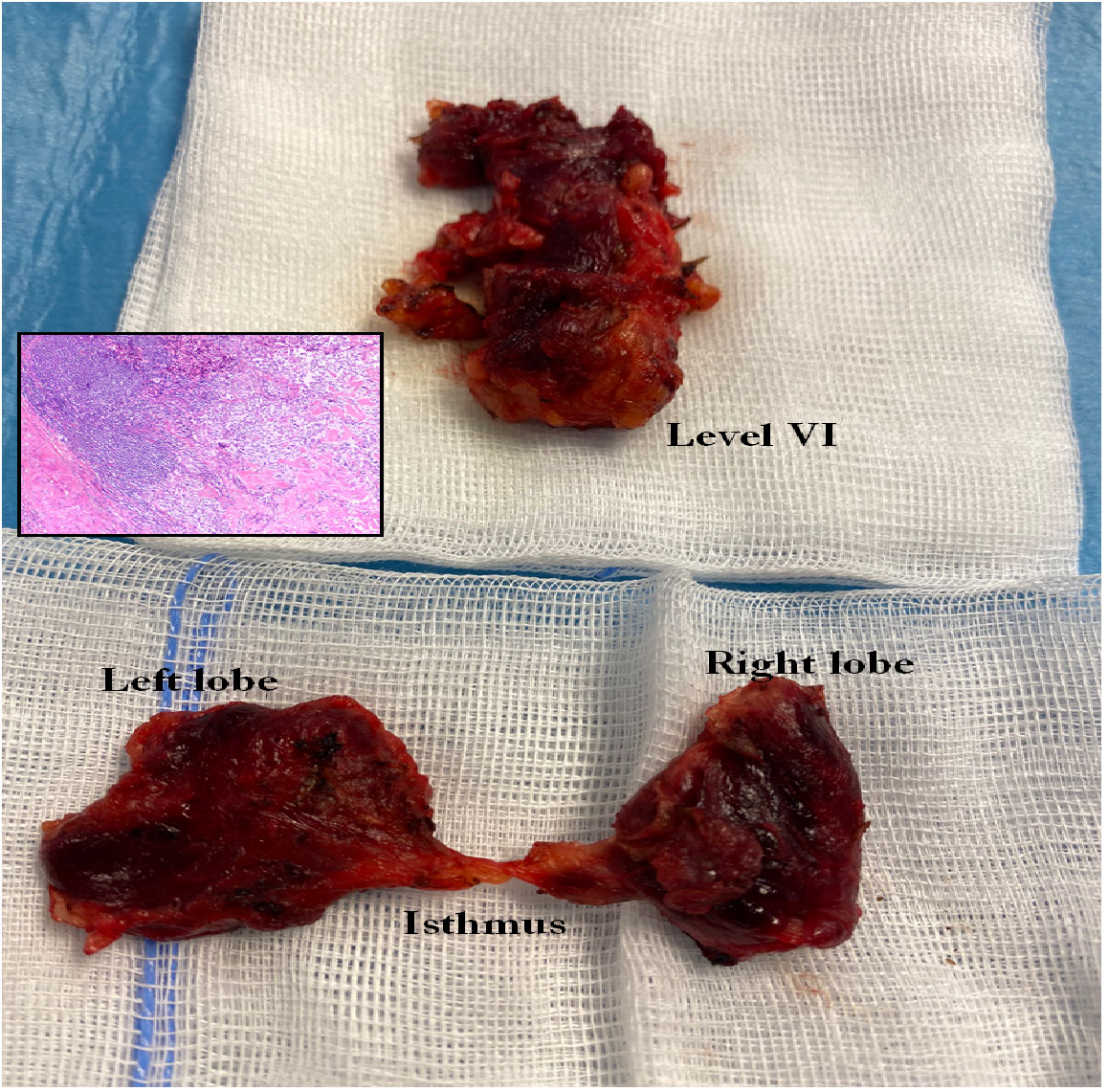
Total thyroidectomy and lymphadenectomy of the central compartment (level VI and VII) in MTC.

The primary therapy for MTC is surgery. Total thyroidectomy is the minimum procedure, depending on the preoperatively measured Ctn and the tumour stage, supplemented by a central, possibly lateral, unilateral/bilateral lymph node dissection (LN dissection) (**Figure 3**). In the case of an MTC discovered incidentally in the histology, the postoperative Ctn value should determine the further procedure: If the Ctn value is not measurable, no further surgery is necessary; if the Ctn value is elevated, supplementary surgery (total TX and central and lateral lymph node dissection) should be performed depending on the tumour stage. Surgical curative therapy is only possible in this situation if no distant metastases and no infiltration into the soft tissue have been described in the primary histology and

- If < 10 affected cervical lymph nodes in the previous histology or
- < 3 affected compartments are detectable after systematic lymphadenectomy (16).

If this favourable situation is present, curative surgical treatment should be attempted. In the case of hereditary MTC, total thyroidectomy is always advisable, as in principle any C-cell can develop into an MTC. In patients with MEN2 detected in family screening, the timing of “prophylactic thyroidectomy” depends on the RET mutation and the Ctn value (**Table 1**). With early diagnosis and complete surgery, the disease-specific 5-year survival rate for all MTCs was increased to 89% (17).

## Morbidity of prophylactic thyroidectomy

A prophylactic (or preventive) thyroidectomy is the removal of the thyroid gland in a patient who is still healthy but carries certain genetic mutations that lead to an increased risk of MTC. This option should be carefully discussed with each patient, taking into account the patient’s age, disease risk, expected benefit, potential side effects and psychological impact, and surgical morbidity. It is considered in all major cancer prevention guidelines. Reducing the risk of disease through surgery is an individualised decision that can be made after a careful and thorough assessment and discussion of the person concerned with a team of professionals. This team includes the endocrinologist, the geneticist to discuss the uncertainty of the risk associated with the genetic mutations, the endocrine surgeons to clarify both the type of surgery and the procedure (endoscopic/robotic/traditional) and the recovery options, and the psycho-oncologist to discuss the impact of this decision on the psyche. Despite all the guidelines, the decision to reduce the risk of disease through surgery to remove the target organ of the cancer is by no means ‘zero’ morbidity. Even if it is a preventive operation, the patient must be informed about all the complications of thyroid surgery. There is no such thing as an operation without complications. The operation must be performed by experienced surgeons. The operation must be performed in specialised centres that have all available technologies to reduce and prevent complications (neuromonitoring, autoflorescence).

## Aftercare planning

The success of the primary procedure can be assessed on the basis of the postoperative Ctn value. With regard to the response to primary therapy and prognosis, 3 risk groups of patients can be defined: (i) excellent (Ctn not measurably low), (ii) biochemically incomplete (Ctn measurable, but no tumour tissue detectable) and (iii) structurally incomplete (Ctn significantly increased, evidence of metastatic lymph nodes or distant metastases) (**Table 2**). Depending on the classification, risk-adapted follow-up is performed with calculation of the tumour marker doubling time (i.e. Ctn/CEA) and imaging to detect possible tumour growth and, if necessary, adjustment of the risk group (18, 19).

**Table 2:** Risk-stratified follow-up.

Risk group	Biochemistry	Time interval of follow-up	Imaging	Therapy
*Excellent*, *biochemically cured*	Ctn < 10 pg/ml, CEA normal	12 months	Sonography neck	No
*Biochemical* *incomplete*	Ctn > 10-150 pg/mL, Ctn DT > 24 months	6 months Ctn, CEA, 12 months of imaging	Sonography neck/abdomen, CT mediastinum/lungs, MRI liver	Mostly none, at “stable disease”: "wait and see"
*Structurally incomplete*	Ctn/CEA increased to > 150 pg/ml, DT < 6 months	6 months Ctn, CEA, 12 months imaging, for Progress every 6 months	sonography neck/abdomen, CT mediastinum/lungs, MRI liver	In case of symptoms or progress local and/ or systemic Therapy, TKI

Ctn, calcitonin; DT, doubling time; TKI, tyrosine kinase inhibitor therapy; CT, computed tomography; MRI, magnetic resonance imaging.

**Figure 4:**
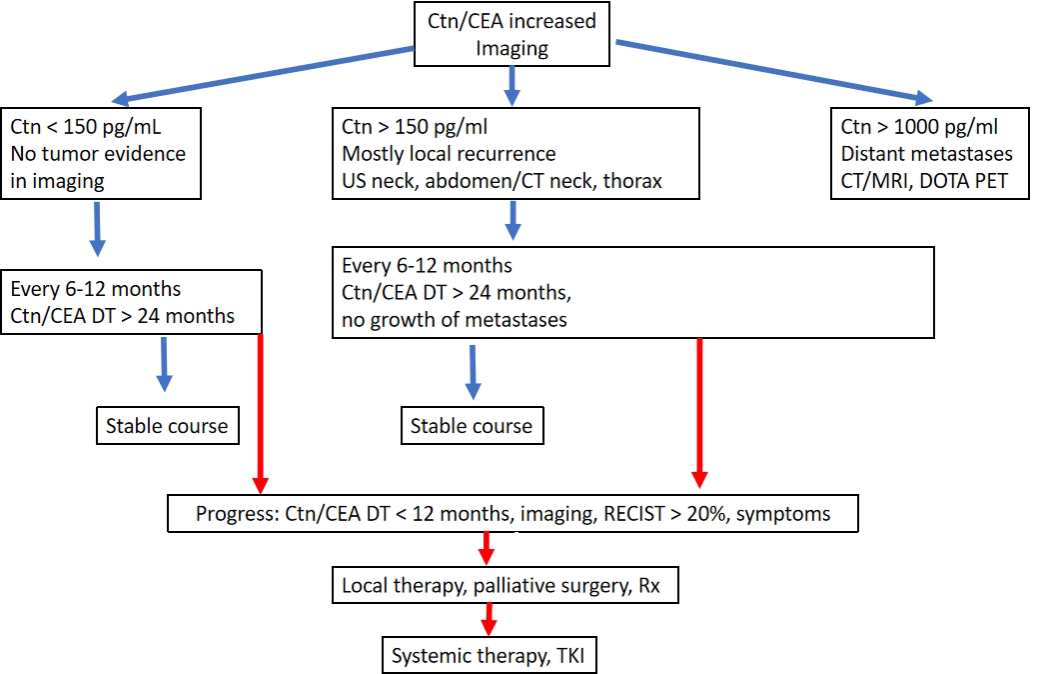
Algorithm for the follow-up of MTC.

The follow-up intervals are risk-adjusted and are performed every 3 months to once a year, depending on the size and location of the residual tumour/metastases and the extent of tumour progression (20). Imaging procedures include sonography of the neck and abdomen, computed tomography with contrast agent, MRI, bone scintigram and possibly FDG-PET or F-DOPAPET, depending on the location of the suspected metastases (**Figure 4**).

Symptoms of recurrent or persistent disease and Ctn and CEA levels drive the indication to perform further imaging procedures. According to ATA guidelines (20) neck ultrasonography should be performed in all of the patients with MTC at follow-up. CT scan of the neck and the chest and a three-phase CT scan of the liver or a MRI of the liver and the axial skeleton are recommended in extensive neck disease or in presence of symptomatic regional or distant metastases, and in all patients with Ctn > 500 pg/mL. If the postoperative serum Ctn level exceeds 150 pg/mL patients should be evaluated by imaging procedures, including neck US, chest CT, contrast-enhanced MRI or

three-phase contrast-enhanced CT of the liver, and bone scintigraphy and MRI of the pelvis and axial skeleton.

In asymptomatic patients with detectable Ctn levels CT o MRI of neck, chest, liver and axial skeleton are advisable. If these imaging exams resulted negative FDG-PET/TC or F-DOPAPET/CT may be indicated considering the doubling time of the markers.

FDG PET/CT and F-DOPAPET/CT proved superior to conventional imaging procedures in detecting metastases in patients with MTC. F-DOPA PET/CT had a higher sensitivity, compared to FDG-PET/CT, and seemed more important in assessing extent of disease. FDG–PET–CT is not recommended for MTCs staging, but it can be useful for assessing advanced disease characterized by dedifferentiation and rapid progression (21).

## Excellent response to the primary therapy group

The Ctn value is not measurable: the patient can be considered biochemically cured if the histology is correct (Ctn immunohistology mandatory). In the first 2 years, six-monthly follow-up examinations with sonography of the neck, Ctn and CEA determination, review of substitution treatment with thyroxine (target: TSH in the normal range), if necessary administration of calcium and calcitriol in the case of postoperative hypoparathyroidism (target: serum calcium in the lower limit range of 2.0–2.3 mmol/l) are sufficient (22). Thereafter, if there is no increase in Ctn, annual checks can be carried out. The prognosis is excellent and does not differ from that of the general population.

## Biochemically incomplete group

The Ctn level is persistently low to moderately elevated (normally < 1000 pg/ml). It can be assumed that residual tumour tissue is present. If the previous operation was inadequate, another suitable operation is performed after staging. If the tumour has progressed beyond the above-mentioned limits (see MTC surgery), a curative approach is no longer possible, so that all further therapeutic measures should be weighed up in terms of the risk of morbidity and taking into account the usually good quality of life associated with the tumour disease. Despite an intensive search, it is often not possible to find a reliable tumour correlate when Ctn is only moderately elevated. In the literature (1), it is assumed that at Ctn values < 150 pg/ml, the rest of the detectable tumour tissue is detected using imaging techniques. The progression of the tumour disease can vary greatly over time and can be estimated relatively well based on Ctn and CEA doubling time (https://www.thyroid.org/professionals/calculators/thyroid-cancer-carcinoma/). At least 4 Ctn values over 2 years are a prerequisite for a good statement. In patients with tumour marker doubling times < 24 months had progression in 94%, also based on image morphology. If tumour marker doubling times > were 24 months, 86% of patients had no detectable tumour growth (23).

In the further course, six-monthly follow-up checks are usually sufficient. The frequency of diagnostic imaging can be planned depending on the primary findings, growth over time (RECIST criteria) and the tumour marker doubling time. Close-meshed maximum diagnostics without therapeutic consequences is not advisable, e.g. sonography every 6 months, computer tomography/MRI every 12 months.

## Structurally incomplete group

The Ctn level is significantly elevated (usually > 1000 pg/ml): Local infiltrating tumour tissue or distant metastases, usually in the lungs, liver and/or bones, can be assumed here. A curative approach is no longer possible; palliative measures take centre stage (24).

## Local therapy of locoregional recurrence and distant metastases

Reoperations with a palliative approach are particularly useful in cases of progressive local recurrence or painful lymph nodes in the central neck area (near the trachea or oesophagus/infiltration of the recurrent laryngeal nerve) in order to reduce local complications. It is not advisable to operate immediately on every newly discovered neck metastasis, as this leads to numerous ineffective operations that do not affect the overall course and are often associated with side effects such as recurrent paralysis, hypoparathyroidism and paralysis of the arm muscles. Radiotherapy is helpful for inoperable local or mediastinal recurrences or painful bone metastases. As surgery is more difficult after radiotherapy, operability should always be considered from a palliative point of view.

Liver metastases are common, usually cause little discomfort and are rarely an indication for surgery. Liver metastases that are significantly advanced and painful should be treated. As a rule, these are multiple and diffuse metastases. Local therapy using (chemo)embolisation or selective internal radiation therapy (SIRT) is only partially effective. Systemic therapy with tyrosine kinase inhibitors is useful if the disease progresses rapidly. Bone metastases are rarely osteolytic, fracture-prone or painful. External radiotherapy is appropriate for painful bone metastases, external radiotherapy or surgery for fracture risk. There are no evidence-based studies on bisphosphonate/denosumab therapy for MTC. In analogy to other tumour diseases, these therapies would also be used individually, especially in cases of pain or fracture risk, particularly in osteolytic metastases (5, 25–27).

## Systemic therapy with tyrosine kinase inhibitors

The usual form of therapy, chemotherapy, is only partially effective in metastasised MTC. Tyrosine kinase inhibitors (TKIs) have been tested in trials for advanced metastasised MTC for around 10 years. The tyrosine kinase inhibitor vandetanib (Caprelsa^®^) has been available for the treatment of aggressive and symptomatic MTC since 2012, and cabozantinib (Cometriq^®^) has been approved for progressive MTC since October 2014. As there is no clear evidence of benefit in early stages of the disease, treatment with TKIs is currently indicated for high tumour burden and progressive (RECIST criteria, short tumour marker doubling time) as well as pronounced symptomatic disease when local treatment measures have been exhausted. To date, no TKI has been shown to prolong survival in metastasised MTC (4, 15, 28).

## Follow-up care for MEN2

In addition to the MTC follow-up examination, pheochromocytoma diagnosis by metanephrine and catecholamine determination is performed annually from the age of 11 years for high and highest risk RET mutations and from the age of 16 years for moderate risk RET mutations and other mutations, and MRI imaging is performed if necessary. In primary hyperparathyroidism, annual follow-up is performed with determination of serum calcium and parathyroid hormone (**Table 1**) (28–30).

## Conclusion

Medullary thyroid carcinoma is a rare neuroendocrine tumour characterized by some atypical and controversial issues. The diagnosis of the primary tumour and of recurrent tumour is challenging. Dosage of Calcitonin and CEA has an important role in the diagnosis and prognosis, in relation to imaging exams. Calcitonin presents high sensitivity but mild specificity and CEA levels increase in relation to the burden of the tumour. Levels of Ctn are functional in the diagnosis of primary MTC and/or MTC relapse detection. Interestingly, preoperative values of Ctn and postoperative values of Ctn in the case of an MTC discovered incidentally should determine the extent of surgery, and particularly of cervical lymph node dissection.

These atypical keypoints of MTC make challenging not only the diagnosis of MTC but also the best treatment to adopt for the primary tumour and even more for the recurrences.

Detection of germline mutation of the RET gene in family screening should indicate prophylactic thyroidectomy.

## Author contributions

DZ: Writing – review & editing. NL: Writing – original draft. HS: Writing – review & editing. FF: Writing – original draft. CS: Writing – original draft. MY: Writing – original draft. HW: Writing – original draft. GD: Writing – review & editing.
